# Determining the challenges and opportunities of virtual teaching during the COVID-19 pandemic: a mixed method study in the north of Iran

**DOI:** 10.1186/s13104-024-06806-8

**Published:** 2024-05-27

**Authors:** Aram Ghanavatizadeh, Ghahraman Mahmoudi, Mohammad-Ali Jahani, Seyedeh Niko Hashemi, Hossein-Ali Nikbakht, Mahdi Abbasi, Alameh Darzi, Seyed-Amir Soltani

**Affiliations:** 1 Hospital Administration Research Center, Sari Branch, Islamic Azad University, Sari, Iran; 2 Social Determinants of Health Research Center, Health Research Institute, Babol University of Medical Sciences, Babol, Iran; 3 Doctorate of Medicine, Shiraz University of Medical Sciences, Shiraz, Iran; 4 Department of Health Economics and Management, School of Public Health, Tehran University of Medical Sciences, Tehran, Iran; 5 Babol Education Department, Babol, Iran; 6 Golestan University of Medical Sciences, Gorgan, Iran

**Keywords:** Virtual education, Active learning, Feedback, COVID-19 pandemic

## Abstract

The aim of this study was to determine the challenges and opportunities of virtual education during the COVID-19 pandemic. This study was conducted in 2022–2023 with a mixed method. During the quantitative phase, we chose 507 students from Mazandaran Province medical universities (both governmental and non-governmental) by stratified random sampling and during the qualitative phase 16 experts were collected by purposive sampling until we reached data saturation. Data collecting tools consisted of questionnaires during the quantitative phase and semi-structured interview during the qualitative phase. Data was analyzed using SPSS21 and MAXQDA10. Mean scores of the total score was 122.28±23.96. We found a significant association between interaction dimension and background variables (*P* < 0.001). The most important privilege of virtual education is uploading the teaching material in the system so that students can access the material constantly and the most important challenge regarding virtual education is lack of proper network connection and limited bandwidth. Virtual education proved to be a suitable alternate to traditional methods of medical education during the COVID-19 pandemic in theoretical topics, we recommend that educational policymakers would take the necessary actions to provide the requirements and facilities needed to improve the quality of virtual education.

## Introduction

Given the adoption of technological advances in healthcare in the context of the COVID-19 pandemic, the dissemination of telehealth practices has dramatically increased between 2020 and 2021 [1]. Virtual education serves as a dynamic substitute for learners to constantly continue their educational journey and preserve their goals [2].

Virtual education is defined as the use of computer-based technology to provide education which consists of online learning, offline learning, or a combination of them [3]. In the web-based virtual educational platforms, students can continue to participate in the live academic lectures which they used to attend in class [4]. Virtual education varies significantly from in-person education [5]. The unavailability of necessary infrastructure and effective organizational strategies have been a major challenge for the integration of virtual education and face-to-face education [6]; [7] and this pandemic not only created the need but also provided the opportunity for accelerating digital transformation in medical education [8]. Using virtual education, learners can save and review the lectures whenever and wherever they want to. however, most professors have less experience with virtual teaching [9]. In this educational model, social interactions between the teachers and the students are solely through internet. Yet, how can a professor provide all the necessary communication, guidance, and feedback through internet alone so that learning process would be effective? Moreover, how can professors make sure that all students have access to the same content and same feedback equally? There are so many online educational activities that not all students can access equally due to differences in network conditions and inequal internet access [10]. Internet inequality could be defined as inequal distribution or access to the internet [11]. However, internet-based learning should be adjusted to different educational modalities so it would be effective [12]. Online education has played an important role in the education of undergraduate and graduate level students, and even continuing medical education (CME) of graduate doctors [13]. Learners can participate in the educational classes anytime it suits them [14].

one study conducted in Saudi Arabia revealed that using web-based video conferences during COVID-19 pandemic resulted in medical students’ satisfaction [15]. In another study by Cataudella et al. in Italy titled “Teaching During the COVID-19 Pandemic” showed that teachers exhibited lower self-esteem and self-efficacy while teaching virtually [16]. Rossi et al.’s study in Brazil demonstrated that active learning tools are helpful for students during the pandemic and they have succeeded at improving their critical thinking, motivation, and their contribution to science [17]. Khalili’s study in the United States showed that e-learning is becoming the new norm in the universities and this development can bring challenges to some, because some teachers lack the proper knowledge and expertise to create an supportive, positive and interesting environment to engage their students [18].

Currently the question is whether the implementation of virtual education has been able to satisfy students in their academic progress? Because learners are important stakeholders during the entire teaching and learning process in all educational institutions [19] and learners’ engagement in this process has positive effects on their active learning [20]. Therefore, due to widespread use of virtual education and online teaching during the pandemic, it is necessary to conduct more studies to examine the challenges and opportunities of this type of education during the COVID-19 pandemic.

## Methodology

### Study design

The current study was conducted using a mixed (qualitative-quantitative) method in 2022–2023 in the north of Iran.

### Sample size

The study population in the quantitative phase consisted of 13,500 medical science students of all the medical universities in the province, both public and private, who benefited from virtual education during the COVID-19 pandemic. In the qualitative phase the population included experts and university professors of the same aforementioned universities.

In order to determine the sample size, it is appropriate to use between 5 and 15 observations for each variable measured [21], in this study we considered between 12 and 13 times the number of questions in the questionnaire, that is in the range of 480 and 520 participants.

The sampling method implemented in the quantitative phase was cluster sampling; first the university, then the academic major, and finally the class was considered as the cluster. 507 students were selected. 194 students from Babol university, 225 students from Sari university of Medical Sciences, and 88 students from Azad university of Sari participated in this research. Inclusion criteria in quantitative phase of this study was that participants had to be a student in one of the medical universities of Mazandaran and also to have consent to participate in the study. participants were excluded if they did not use online education methods.

In the qualitative phase we selected 16 academic staff members of both basic science and clinical stage, using purposive sampling, who were policymakers and planners in their universities and had online educational activities alongside their executive posts.

### Information gathering tools

After our proposal was accepted and we obtained the ethics committee approval, we commenced our executive phase of the research. For the literature review all the articles that were published between 2010 and 2021 in different national and international databases including ISI, Pubmed, Scopus, Google Scholar, Magiran, SID, and Irandoc were reviewed. We used Persian and English keywords including online teaching, virtual teaching, active learning, COVID-19, interaction in virtual education, feedback in online education, benefits of virtual learning, disadvantages of virtual learning, and types of virtual learning.

### Data measurement tools

In the quantitative phase we used a questionnaire developed by Ünal Çakiroğlu and colleagues [22]. The questionnaire consists of 7 different principles. Each principle consists of approximately 5–6 questions and a total of 40 questions and answers vary between range of not satisfied to perfectly satisfied and each question has a score of 1 to 5.

In order to convert the English questionnaire to Persian we used the translation-retranslation method as described below. At first, two translator’s experts in this field translated the English version into Farsi. A conceptual translation instead of word-to-word translation was implemented; also, clarity, simplicity, brevity, type of audience, age and cultural factors were taken into consideration by the translators. In the second stage two translators fluent in English, who were not aware of the questionnaire’s content, translated the questionnaire back into English. In general, conceptual similarity was an important factor during the translation process. finally, in the third stage an expert panel consisting of people fluent in both languages reviewed the quality of the translations in the presence of researchers and in the case of inconsistency between translations, alternative words were suggested.

To perform face validity, the questionnaire was given to 20 students (10 males and 10 females) who met the inclusion criteria and a number of related experts. They were asked for feedback about the clarity of the questionnaire, its readability, writing style, easy understanding, confusing words, comprehensibility, disproportion and ambiguity. Any needed corrections were applied.

To check the reliability, Cronbach’s alpha was used as the index to evaluate internal consistency for the entire questionnaire and for each scale. Values above 0.7 were considered acceptable. To evaluate intraclass reliability, we used test-retest method. Data from 30 students, who met the inclusion criteria, were collected in two stages with a time interval of one month then the scores obtained in these two stages were evaluated using intraclass correlation coefficient (ICC).

To perform the qualitative phase, the data collection tool was semi-structured interview. We used the data from the quantitative phase of the study, the quantitative statistical outputs and items in the questionnaire that had the lowest points from the students’ point of view to formulate our interview questions. In the way that less favorable items from the students’ point of view were used as questions in the qualitative phase. At this point we used semi-structured interview and in-depth interview to gather our data. After conducting the interviews, handwriting, typing and listening to the files several times all the notes and writings were named and coded. in the initial coding process, the researcher reviewed the written and typed data line by line as analytical units; and then by identifying the related semantic units or determining the important parts of the text, the researchers would extract the explicit meanings and concepts from the interview texts and would write them next to the relevant sentence in the form of a code. At the same time in another text the researcher wrote down the codes with the relevant address.

### Data analysis

After collecting the data and coding, the results of the quantitative phase were entered into the SPSS21 software. In order to perform the related statistical tests, first, the normality of the data was checked using the Kolmogorov-Smirnov test. In order to check the linear relationships between quantitative variables, Pearson correlation coefficient was used. Friedman’s test was used to rank principals and dimensions at a significance level of *p* < 0.05. For analysis of the qualitative phase, content analysis method and MAXQDA10 software were carried out.

## Results

### Results of the quantitative phase

From the 507 students participating in this study, regarding the demographic characteristics, the mean age of the participants was 21.47 ± 2.34 years with a range of 18 to 43 years. 319(62.92%) females and 174(34.32%) males participated in the study. The majority of the participants 145(28.6%) were medical students. (Table 1)

**Table 1:** Study participants demographics

Variable	Groups	Frequency	Frequency percentage
Sex	Male	188	37.08
	Female	319	62.92
Age group	≤20	149	29.39
	21	174	34.32
	22	98	19.33
	≥23	86	16.96
Grade	Bachelor	279	55.03
	Master	8	1.58
	Doctorate	196	38.66
	Residency	24	4.73
Year	First year	113	22.29
	Second year	161	31.75
	Third year	193	38.07
	Fourth year and above	40	7.89
Academic major	MD	145	28.60
	Public health	73	14.40
	Nursing	70	13.81
	Pharmacology	51	10.06
	Radiology	46	9.07
	Midwifery	35	6.90
	Dentistry	23	4.54
	Others	64	12.62

Descriptive statistics of the scores of principles and dimensions of the questionnaire showed that the mean and standard deviation of the total score is 122.28±23.96 and for the three dimensions of interaction, learning and teaching. They were 34.54±8.23, 33.80±8.01 and 53.93±10.15. (Table 2)

**Table 2:** Descriptive indicators of virtual teaching based on principles and dimensions of active learning according to students of mazandaran province medical universities during the COVID-19 pandemic

Principles and dimensions	Principles and dimensions title	Questions	Score range	Mean (SD)	Median (IQR)	Min	Max
Principles	Student-professor interaction	6	6–30	5.28 ± 17.77	(21 − 14)18	6	30
	Cooperation between students	6	6–30	3.79 ± 16.77	(19 − 14)17	6	28
	Time on activity	6	6–30	4.18 ± 17.87	(20 − 15)18	6	29
	Instant feedback	5	5–25	3.30 ± 16.41	(19 − 14)17	7	24
	High expectations	6	6–30	4.40 ± 19.64	(23 − 17)20	6	30
	Active learning	6	6–30	4.47 ± 18.42	(21 − 16)18	6	30
	Diverse talents and ways of learning	5	5–25	4.12 ± 15.38	(18 − 13)16	5	25
Dimensions	Interaction	12	12–60	8.23 ± 34.54	(40 − 29)35	13	58
	Teaching	17	17–85	10.15 ± 53.93	(60 − 48)54	19	81
	Learning	11	11–55	8.01 ± 33.80	(39 − 28)34	12	55
Total		40	40–200	23.96 ± 122.28	(138 − 106)122	47	194

The results of the test about the correlation of the seven principles of the questionnaire showed that all seven principles had a positive correlation with each other and the correlation was of strong intensity. The strongest relationship was between high expectations and diverse talents (*r* = 0.73, *p* < 0.001). The lowest correlations were found between cooperation among students and the time on task with other principles, the rest of the correlations are provided in the table. (Table 3)

**Table 3:** Evaluation of correlation between principles, students’ point of view on virtual education during the COVID-19 pandemic

Questionnaire principles	Student-teacher interaction	Cooperation among students	Time on task	Prompt feedback	High expectation	Active learning	Diverse talents and ways of learning
Student-teacher interaction	1						
Cooperation among students	^*r*=0.635^ ^< 0.001^	1					
Time on task	^*r*=0.623^ ^< 0.001^	^*r*=0.518^ ^< 0.001^	1				
Prompt feedback	^*r*=0.603^ ^< 0.001^	^*r*=0.511^ ^< 0.001^	^*r*=0.580^ ^< 0.001^	1			
High expectations	^*r*=0.614^ ^< 0.001^	^*r*=0.505^ ^< 0.001^	^*r*=0.543^ ^< 0.001^	^*r*=0.653^ ^< 0.001^	1		
Active learning	^*r*=0.556^ ^< 0.001^	^*r*=0.517^ ^< 0.001^	^*r*=0.509^ ^< 0.001^	^*r*=0.601^ ^< 0.001^	^*r*=0.660^ ^< 0.001^	1	
Diverse talents and ways of learning	^*r*=0.625^ ^< 0.001^	^*r*=0.536^ ^< 0.001^	^*r*=0.605^ ^< 0.001^	^*r*=0.628^ ^< 0.001^	^*r*=0.744^ ^< 0.001^	^*r*=0.739^ ^< 0.001^	1

*Pearson correlation coefficient test- Significance level *P* < 0.05

Also, in examining the relationship between the three dimensions of the questionnaire, the results showed that the highest correlation was between learning with teaching (*r* = 0.786, *P* < 0.001) and the lowest correlation was between interaction with learning (*r* = 0.666, *P* < 0.001), The correlation between teaching and interaction was (*r* = 0.738, *P* < 0.001).

The result of the Friedman test showed that from the participants’ point of view, there was a difference between dimensions of online teaching including interaction, teaching and learning. In other words, according to participants, online teaching during the COVID-19 pandemic had the best performance in teaching and the weakest performance in learning. (Table 4)

**Table 4:** Average score of interaction, teaching and learning dimensions and their principles

Dimensions	Scores	Principles	Scores
Interaction	1.57	Student-teacher interaction	4.20
		Student-student cooperation	3.56
Teaching	3.00	Activity time	4.32
		Prompt feedback	3.22
		Active learning	4.70
Learning	1.44	High expectation	5.49
		Diverse talents and ways of learning	2.51

### The results of the qualitative phase

In the qualitative phase of the research, interview method was used to collect the data. The characteristics of the interview participants are provided in Table 5.

**Table 5:** The information of participating professors in the interview about opportunities and challenges of virtual teaching for active learning

Row	Gender	Specialty	Grade	Position	Scientific ranking	Educational group
1	Male	Nursing	PhD	Faculty Member	Assistant Prof.	Psychiatric nursing
2	Male	Educational management	PhD	Faculty Member	Associate Prof.	Medical education
3	Female	Educational management	PhD	Faculty Member	Associate Prof.	Educational management
4	Male	Software systems	PhD	Faculty Member	Prof.	Computer engineering
5	Male	Teaching philosophy	PhD	Faculty Member	Assistant Prof.	Educational management
6	Female	Public health education and health improvement	PhD	Faculty Member	Assistant Prof.	Public health
7	Female	Health policymaking	PhD	Faculty Member	Assistant Prof.	Public health
8	Male	Social medicine	MD	Head of department and faculty member	Prof.	Social medicine
9	Female	Tropical and infectious disease	PhD	Head of department and faculty member	Assistant Prof.	Clinical medicine
10	Male	Medical virology	PhD	Education deputy and Faculty Member	Associate Prof.	Microbiology
11	Male	Anatomy	PhD	Faculty Member	Assistant Prof.	Anatomy
12	Female	Heart failure and transplant	MD (fellowship)	Faculty Member	Assistant Prof.	Cardiovascular
13	Male	Pathology	MD	Faculty Member	Associate Prof.	Pathology
14	Female	Psychiatry	MD	Faculty Member	Assistant Prof.	Psychiatry
15	Female	Pathology	MD	Faculty Member	Assistant Prof.	Pathology
16	Male	Cardiovascular	MD	Faculty Member	Assistant Prof.	Cardiovascular

In Table 6, there are Strengths, opportunities and challenges of virtual education according to experts. Some solutions were suggested to improve this teaching method.

**Table 6:** Strengths, opportunities and challenges of online teaching and their effect on active learning

Topic	Row	Description	Frequency
Strengths & Opportunities	1	Uploading educational material	16
	2	Increased ability of professors following virtual teaching experience and working with various educational software	16
	3	Availability of some technical infrastructure in universities before the pandemic	16
	4	The ability to upload assignments in audio and video form	16
	5	Specifying assignments	16
	6	Ability to change and edit uploaded assignments	16
	7	Ability to receive uploaded files	16
	8	Ability to conduct examinations in MCQ and descriptive form	16
	9	Constant access of students to educational material	16
	10	Different methods of sending and receiving feedback	15
	11	Feedback from professors to students	14
	12	The possibility of online teaching during shutdown of classes	14
	13	The possibility of increased teaching time in theoretical topics	13
	14	Lower costs for professors and students due to less commute time	13
	15	Improving the technical infrastructure of universities and medical training centers following the advent of virtual teaching	13
	16	No restriction on education due to the geographical distance of students from the university where they study	13
	17	Ease of access through social networks	12
	18	Increased teaching time due to educational unit supervision on the teaching of professors through the system	12
	19	Continuous education	12
	20	Participation in scientific seminars of various domestic and foreign universities without paying travel expenses	11
	21	Conversation in written or audio format	11
	22	The possibility of obtaining a premade educational package of videos in the university library	7
	23	Peace of mind following learning and teaching at the place of residence	7
Challenges	1	Lack of proper communication infrastructure (internet)	16
	2	Less interaction in online teaching	16
	3	Online test conduction	16
	4	Lack of proper skills in professors to hold online classes	15
	5	Decreased student participation	14
	6	Professors’ dissatisfaction with students’ participation	14
	7	Lack of control over students	14
	8	Passive attendance in classes	12
	9	Increased personal expenses	11
	10	Lack of appropriate hardware for this teaching method by professors and students	11
	11	Incompetence of existing platforms to handle the number of students using these platforms during the pandemic	10
	12	Holding practical classes	8

### Qualitative phase data analysis

One of the most important opportunities of virtual teaching according to experts is the possibility of uploading the educational material in the electronic domains. The most important challenge of virtual teaching was the lack of proper and desirable communication infrastructure (internet) and poor-quality internet. The first solution proposed by the professors to increase the quality and efficacy of online teaching is recognizing the problems related to this educational method. the most important influential weakness of this method was the lack of proper communication and internet infrastructure.

## Discussion

The results of the study showed that all the principles of the study were positively correlated to each other and this relation was strong. The strongest relation was between high expectations and diverse talents and also between active learning and diverse talents. The least correlation was between cooperation among students and time on activity with other principles. In a study conducted by Alahmadi et al. it was shown that students believe online classes help them overcome some learning obstacles for example the fear of communication in English. One can argue that virtual classes are helping learners, especially timid learners, to interact more and overcome their fears of interaction in face-to-face classes [23]. Tanis article revealed that quick interaction between peers is helpful for their learning, whereas isolation and lack of communication was harmful, However, group project was not the best way of learning. Students found delayed feedback and limited work by their peers harmful for learning and preferred to work at their own pace [24].

In investigating the relationship between different principles, the strongest relationship was between teaching and learning and the least relation was between interaction and learning. Alenezi study mentions that it is necessary to design an effective electronic learning environment in which the content is presented based on the characteristics of the teachers and learners, the structure of the educational material and interaction creation [25]. In another study by Adnan et al. inaccessibility to internet, lack of proper interaction among teachers and learners and inefficient technology were the major challenges of students [26].

The results of the Friedman test in the present study showed that there are differences between various dimensions of virtual teaching including interaction, teaching and learning. In other words, online teaching has had the best performance in the teaching dimension and the weakest in the learning dimension during the COVID-19 pandemic. Çakiroğlu observed in his study that although the electronic learning system has advantages, there are still challenges related to the cooperation between students, and active learning was very low in virtual education [22]. Other studies showed that active learning using simulations improves conceptual learning and memory, increases motivation and study intensity, and also reduces the achievement gap in basic students [27, 28].

According to the results of the study, one of the most important opportunities of virtual teaching is the ability to upload the teaching material in the electronic system, because it enables the students to receive and use the material as many times as they need to; this will eventually result in improvement of learning quality provided that we address the issues and challenges related to this process [29]. In electronic teaching, educational content can be quickly delivered to learners and standardized and updated if necessary. The material can be delivered using different approaches including self-directed and coaching [30].

Another strong point in online and virtual education was the increased ability of professors in working with different educational software, uploading assignments, holding online exams, and learning different ways of feedback. In Bjekic’s study, a teacher’s capability in electronic teaching is considered a combination of educational, communicational and content creation capability [31] and teacher’s ability to conduct better tests results in students’ satisfaction [32, 33]. In this regard the faculty can use both online and offline tools for the education of students [34]. However, we should keep in mind that medical education is not solely the theoretical matters, there are also other aspects of teaching including laboratory techniques, clinical skills and patient exposure; so electronic methods alone will not be sufficient for medical education [35].

One of the other opportunities provided by virtual teaching is decreased expenses of both teachers and students due to less commute and the easy access of students to their professors through social network and also lack of limitations because of geographical distance between one individual’s residence and the place of their institute, Fedynich states that one of the advantages of remote education is that it is not limited by the learners’ location [36], also, it can be provided by the professors regardless of their location [37], it decreases students’ expenses [33, 38] and students can ask questions whenever they have trouble with their studies and receive answers in a short amount of time and they can also see questions asked by others [39].

Of the important challenges regarding virtual education, we can mention the lack of proper communication infrastructure (internet) and low-quality internet which can affect the quality of online classes as well as examinations. An important weakness of virtual teaching is inaccessibility to digital products needed for the education by the students [40]. Not having reliable internet connection [41, 42], hardware and software issues of virtual educational platforms [43, 44], problems related to speed and quality of internet [45, 46] and audio and video streaming issues are other disadvantages of this teaching method [47].

Another engagement of professors in online education is the topic of conducting online examinations, because they believe online platforms do not hold the capacity to perform reliable and valid examinations, the results of several studies showed that due to the lack of supervision during the test, students’ grades are significantly higher compared to their previous educational records [37, 48, 49]. On the contrary, in Lara et al.’s study scores recorded from 49 medical students in OSCE did not have a significant difference from scores obtained from the same exam conducted in-person [50], it seems like there is a difference between the nature of theoretical and applied examinations.

The other challenge was organizing practical courses and clinical rotations. Due to several infrastructural and human limitations, holding online practical classes was not possible, and students passing their clinical rotations or those who had applied courses faced many problems. In this regard, the main issue is due to the very nature of medical education and the main problem is the inability to practice and obtain clinical skills online [51]. Clinical courses have suffered from the suspension/reduction of undergraduate student internships with a knock on impact on education. The fulfilment of professional skills in clinical training present both educational and professional challenges. Medical teachers will need to innovate and think outside the box to maintain the value of medical education in extreme circumstances. A solution may be represented and the introduction of telemedicine technologies that may contribute to the improvement of core competencies, medical knowledge, overall learning, and higher quality patient care [52]. We should keep in mind that online education cannot replace the face-to-face education of laboratory skills and techniques [53]. And most students do not feel good about learning practical skills alone or online [39].

### Study limitations

This study was limited to Mazandaran Province. Also, the study population was limited to the students of the Medical Sciences universities. On the other hand, we evaluated all academic levels together, and considering the fact that challenges are different in various stages of training and in different majors this may affect the overall results. For example, students in their clinical rotations have very different problems than those passing theoretical courses and topics.

## Conclusion

Since virtual education proved to be a suitable replacement for traditional educational methods in theoretical subjects during the COVID-19 pandemic and considering the recognition of factors affecting the quality of virtual teaching; it is crucial for policymakers in the field of education to take these factors into consideration, and implement goal-oriented plans and do their best to provide the necessary requirements to improve the quality of virtual teaching, so that it ultimately leads to an increase in the quality of learning of medical students.

## Data Availability

No datasets were generated or analysed during the current study.
